# Comparative analysis of psychological competitive abilities in South Korean, Chinese, and Japanese youth soccer players

**DOI:** 10.3389/fpsyg.2026.1415774

**Published:** 2026-04-02

**Authors:** Changliang Yan, Jungwoon Seo, Yongse Kim, Qingyun Jin

**Affiliations:** 1 Department of Physical Education, Northeastern University, Shenyang, China; 2 Dongguk University Seoul, Seoul, Republic of Korea; 3 Korea Institute of Sport Science, Seoul, Republic of Korea; 4 Ningbo University, Ningbo, China

**Keywords:** China, Japan, psychological competitive abilities, South Korea, youth soccer player

## Abstract

This study analyzed and compared the psychological competitive abilities of 466 youth soccer players from South Korea (*n* = 152), China (*n* = 151), and Japan (*n* = 163) using the Diagnostic Inventory of Psychological Competitive Ability (DIPCA.3) as the measurement tool. The results revealed significant cross-cultural differences: Chinese players exhibited greater patience (M = 4.42) than South Korean (M = 4.09) and Japanese players (M = 3.94, *p* < 0.001). South Korean and Japanese players showed significantly higher volition for winning (M = 4.31 and 4.37, respectively) than their Chinese counterparts (M = 3.94, *p* < 0.001). South Korean athletes also reported the highest levels of confidence (M = 4.29, *p* < 0.001) and cooperation (M = 4.73, *p* < 0.001). These findings highlight the need for culturally tailored psychological interventions to optimize coaching strategies across East Asian soccer academies.

## 1 Introduction

Achieving peak performance in sports, particularly soccer, demands more than just physical prowess. The intricate interplay of psychological competitive factors significantly influences athletes’ performances and the outcomes of their games (Hadi et al., 2011). Recognizing and harnessing these psychological elements are paramount in the training of soccer players, as they are pivotal predictors of both current and future success (Feichtinger and Höner, 2014; Höner and Feichtinger, 2016; Musculus and Lobinger, 2018; Vaeyens et al., 2008). Psychological skills training (PST), the systematic cultivation of athletes’ psychological competitive abilities, traces its origins to the seminal research of psychologist Griffith (1930) (Tokunaga, 2001). These abilities, encapsulated in the concept of psychological competitive ability (PCA), encompass a spectrum of psychological factors and traits that empower athletes to perform optimally under competitive pressure (Tokunaga, 2001). The burgeoning recognition of psychology’s indispensable role in soccer performance is evident across professional leagues worldwide. Clubs such as the Derby County Football Club in the English Premier League and AFC Ajax in the Netherlands exemplify this trend, integrating psychological competitive evaluations into their player development strategies (Raglin, 2001; Vestberg et al., 2012). Moreover, initiatives such as the program administered by the German Youth Soccer Academy underscore a concerted effort to enhance youth athletes’ psychological competitive attributes through periodic evaluations and targeted interventions (Musculus and Lobinger, 2018). Research by Nieuwenhuis et al. (2002) underscores the profound impact of psychological competitive abilities on soccer players’ performance outcomes. As the understanding of psychology’s pivotal role in sports performance continues to evolve, coaches increasingly rely on insights into players’ psychological profiles and competitive history to optimize team performance (Nieuwenhuis et al., 2002). In the present study, we specifically examine and compare the psychological competitive abilities of youth soccer players from South Korea, China, and Japan. Although the importance of psychological skills in soccer performance has been widely acknowledged, limited empirical research has directly compared these attributes across East Asian youth populations within similar developmental stages. Given the shared cultural heritage and distinct training systems across these countries, a cross-national comparison may provide valuable insights into how cultural and developmental contexts shape psychological competitive profiles. By focusing on adolescent players in structured high school programs, this study aims to offer empirically grounded evidence to inform culturally responsive psychological skills training in youth soccer.

Understanding the psychological competitive abilities of soccer players is essential for tailoring coaching and game strategies to maximize performance (Abdullah et al., 2016). In particular, for youth athletes, these abilities are crucial for their transition to adulthood and success in their athletic careers (Morris, 2000). Adolescence marks a pivotal period for cognitive, emotional, and psychological development, during which psychological factors significantly influence interpersonal skills and self-identity (Patel et al., 2007).

To this end, a comprehensive understanding of players’ psychological competitive abilities is paramount. This knowledge enables coaches to anticipate performance outcomes and address potential challenges effectively (Abdullah et al., 2016). Although numerous studies have explored the impact of psychological variables on football performance, there remains a gap in understanding the unique psychological traits of Asian football players, which are influenced by cultural nuances and regional dynamics (Gledhill et al., 2017; Ivarsson et al., 2020; Saward et al., 2020). In contrast to Western contexts, Asian athletes exhibit distinct psychological characteristics shaped by cultural values and societal norms (Babbitt, 2019; Li et al., 2015). For instance, despite sharing Confucian values, South Korean, Chinese, and Japanese athletes demonstrate divergent attitudes toward authority and interpersonal dynamics within their training environments (Lee and Baek, 2008). Cultural differences also manifest in motivation for sports participation, with varying emphasis on internal satisfaction, social values, and individual achievement across countries (Choi, 2011). Recognizing these cultural nuances is imperative for coaches who aim to optimize training methodologies and performance outcomes among diverse athlete populations (Lee and Kim, 2005; Yoon and Jeon, 2011). However, despite substantial investments in soccer across Northeast Asian countries, limited research has compared the psychological competitive abilities of youth soccer players between these nations. As soccer continues to thrive in South Korea, China, and Japan, there is a growing need to deepen our understanding of the psychological factors influencing player performance in these regions (Manzenreiter and Horne, 2007).

This study aims to fill this gap by analyzing and comparing the psychological competitive abilities of South Korean, Chinese, and Japanese youth soccer players. By examining foundational data, we aim to provide coaches with insights that can enhance their training and game strategies, taking into account the cultural influences on athletes’ psychological profiles.

These insights are expected to support youth coaches in tailoring psychological skills training according to players’ cultural backgrounds. For instance, enhancing volition in Chinese athletes or leveraging cooperation tendencies among South Korean players could inform personalized coaching approaches.

Based on previous findings highlighting the influence of cultural and training environments on psychological attributes (Lee and Kim, 2005; Ivarsson et al., 2020), we hypothesize the following:

*H*1: Chinese youth soccer players will score higher on patience.

*H*2: South Korean and Japanese players will show greater volition for winning.

*H*3: South Korean players will demonstrate higher confidence and cooperation than their counterparts.

## 2 Materials and methods

### 2.1 Measurement tools

The Diagnostic Inventory of Psychological Competitive Ability (DIPCA.3) was developed by Tokunaga (2001) using data from 5,334 elite athletes who were assessed for their psychological competitive abilities from 1986 to 1999. In this study, we adapted the DIPCA.3 instrument as revised by Kosaka et al. (2016). The DIPCA.3 consists of 52 items across 12 subfactors—patience, aggressiveness, volition for self-realization, volition for winning, self-control, ability to relax, concentration, confidence, decision-making ability, predictive ability, judgment, and cooperation—with each subfactor containing four items. The inventory comprises 48 items, of which 14 are reverse-scored and 4 are false items included to ensure that participants faithfully complete the questionnaire. Respondents use a 5-point Likert scale to answer the questionnaire’s items, where 1 = strongly disagree and 5 = strongly agree. The internal consistency (Cronbach’s *α*) of each subfactor was as follows: Patience = 0.765, aggressiveness = 0.699, volition for self-realization = 0.600, volition for winning = 0.627, self-control = 0.662, ability to relax = 0.713, concentration = 0.719, confidence = 0.769, decision-making ability = 0.720, predictive ability = 0.722, judgment = 0.802, and cooperation = 0.775. The overall internal consistency of the DIPCA.3 was. 924, confirming an adequate level of reliability.

### 2.2 Participants

We used a simple random sampling method to collect data. Data were collected from a total of 673 participants, comprising 230 South Korean, 194 Chinese, and 249 Japanese male youth soccer players registered with their respective soccer associations. To ensure consistency and comparability across samples and to allow a more accurate comparison of their psychological competitive abilities, we applied strict inclusion criteria. Although 673 responses were initially collected, 197 were excluded because the players did not meet the standardized age range of 16–19 years (e.g., several of the respondents were 15 or 20 years old) or did not belong to top-tier high school or professional teams in their respective countries. Given the sharp differences in developmental and training environments between amateur and elite youth soccer players, and the fact that the three national systems have comparable graduation timelines for high school athletes, we treated the non-conforming data as inappropriate for valid cross-national comparison. Consequently, we focused on a final sample of 466 athletes who met the criteria for age, playing level, and career length. This approach ensured a more homogeneous sample, improving the internal validity of the psychological comparisons (Table 1).

**Table 1:** Characteristics of study participants (*N* = 466).

Nation	Age		Career (year)	
	M	SD	M	SD
South Korea (*n* = 152)	18.24	1.24	7.24	1.79
China (*n* = 151)	17.92	1.62	6.41	2.77
Japan (*n* = 163)	16.48	0.93	10.02	2.18

M, Mean; SD, Standard deviation.

### 2.3 Research process

Ethical approval for this study was obtained from the Dongguk University Research Ethics Committee (approved number DUIRB-202109-22). The DIPCA.3 questionnaire was selected after consultation with two youth soccer coaches to ensure content validity in measuring youth soccer players’ psychological competitive abilities. The questionnaire, originally available in Japanese and English, was translated to Korean and Chinese following the guidelines proposed by the International Test Commission to establish equivalence across the translated versions. Subsequently, the questionnaire was modified to suit the context of youth soccer players. To ensure comprehension and validity, the questionnaire was reviewed by individuals with expertise in sports psychology and proficiency in the respective languages and cultures. This panel included a sports psychologist with over 25 years of experience in China, a bilingual expert with knowledge of Japanese, Korean, and English, a South Korean sports psychologist, a South Korean sports sociologist, and two South Korean youth soccer coaches with more than 5 years of experience. Upon finalizing the questionnaire, current youth soccer coaches in South Korea, China, and Japan were contacted for participation. Coaches who consented to participate were provided with detailed explanations of the study’s purpose and methodology and were asked to distribute the questionnaires to their players. In light of the COVID-19 pandemic, the questionnaires were delivered to teams via both mail and electronic means, with coaches facilitating the distribution to players. Prior to data collection, the players were briefed on the questionnaire’s purpose and instructions. Coaches were instructed not to assist the players in completing the online questionnaires. The first page of the questionnaire included a summary of the study’s objectives and assured participants that the collected data would be used solely for academic research purposes and in accordance with research ethics regulations. Data collection occurred from 1 March 2021 to 31 May 2021, involving 466 male football players across South Korea (152), Japan (163), and China (151). The DIPCA.3 questionnaire was distributed online to coaches, who then disseminated the questionnaire links to their players.

### 2.4 Data analysis

Data were analyzed using SPSS 20 software. First, participants’ characteristics and normality of the parameters were assessed using frequency analysis and descriptive statistics. Second, the reliability of the questionnaire was verified based on internal consistency. Third, differences in psychological competitive abilities among South Korean, Chinese, and Japanese youth soccer players were analyzed using analysis of covariance (ANCOVA), with age included as a covariate to control for its potential effect. The homogeneity of variance for each dependent variable was tested using Levene’s test. If the significance probability of Levene’s test was greater than 0.05, *post-hoc* comparisons were conducted using Bonferroni correction. If the significance probability of Levene’s test was lower than 0.05, Dunnett T3 correction was applied. Statistical significance was set at a *p*-value of < 0.05.

## 3 Results

### 3.1 Descriptive statistics

**Figure 1:**
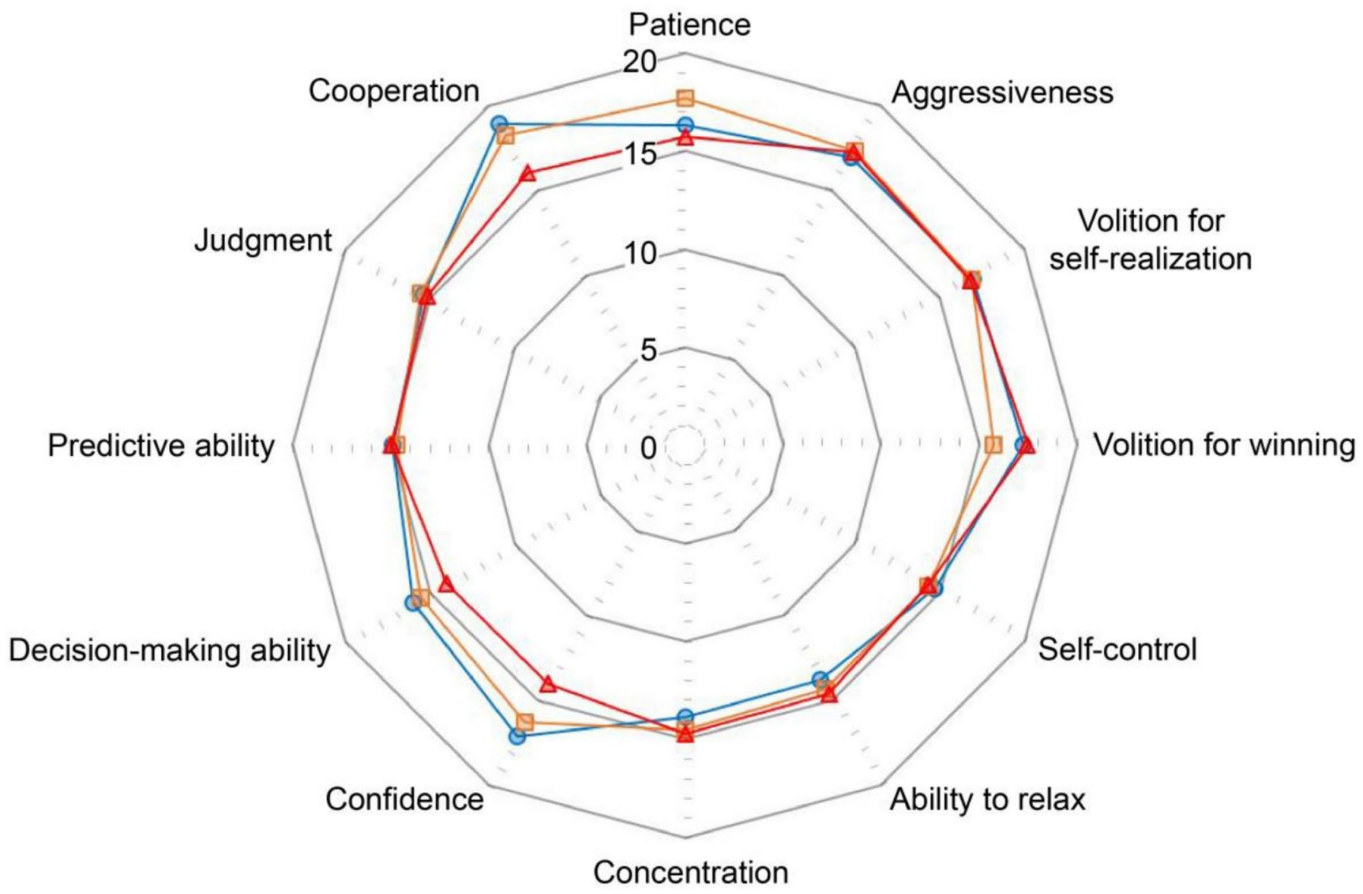
The profiles by subscales of psychological competitive abilities for South Korean, Chinese, and Japanese youth soccer players.

Baseline data were analyzed using descriptive statistics, including the mean and standard deviation (Table 2). The normality of the study parameters was assessed using skewness and kurtosis, and the values did not exceed the criteria (skewness ≤ 2, kurtosis ≤ 4), thereby satisfying the assumption of normality (Hong et al., 2003). The profiles by subscales of psychological competitive abilities for South Korean, Chinese, and Japanese participants are shown in Figure 1.

**Table 2:** Descriptive statistics of measured variables.

Factor	South Korea			China			Japan		
	M (SD)	Skewness	Kurtosis	M (SD)	Skewness	Kurtosis	M (SD)	Skewness	Kurtosis
a	4.09 (0.525)	−0.095	−0.737	4.42 (0.536)	−0.502	−0.905	3.94 (0.815)	−1.025	1.454
b	4.24 (0.533)	−0.507	−0.493	4.33 (0.550)	−0.612	−0.212	4.31 (0.770)	−1.320	1.552
c	4.24 (0.571)	−0.517	−0.396	4.22 (0.540)	−0.420	−0.487	4.20 (0.793)	−1.362	2.367
d	4.31 (0.512)	−1.072	1.457	3.94 (0.683)	−0.672	−0.305	4.37 (0.661)	−1.589	2.431
e	3.67 (0.453)	−1.055	2.531	3.58 (0.839)	−0.214	−0.581	3.57 (0.903)	−0.526	−0.150
f	3.45 (0.887)	−0.080	−0.512	3.59 (0.811)	−0.177	−0.675	3.66 (0.979)	−0.704	0.007
g	3.47 (0.811)	−0.208	−0.056	3.63 (0.848)	−0.225	−0.716	3.68 (0.922)	−0.768	0.085
h	4.29 (0.419)	−0.028	−0.059	4.08 (0.629)	−0.269	−0.535	3.50 (0.864)	−0.175	−0.428
i	4.01 (0.653)	−0.210	−0.759	3.88 (0.608)	−0.073	−0.098	3.51 (0.763)	−0.300	0.357
j	3.72 (0.622)	0.359	−0.478	3.68 (0.609)	0.104	−0.011	3.74 (0.789)	0.040	−0.973
k	3.85 (0.642)	0.113	−0.829	3.89 (0.628)	0.018	−0.527	3.80 (0.812)	−1.051	1.051
l	4.73 (0.292)	−1.375	2.438	4.57 (0.449)	−0.983	0.396	4.02 (0.790)	−0.804	0.379

a = Patience, b = Aggressiveness, c = Volition for self-realization, d = Volition for winning, e = Self-control, f = Ability to relax, g = Concentration, h = Confidence, i = Decision-making ability, j = Predictive ability, k = Judgment, l = Cooperation.

### 3.2 ANCOVA results for psychological competitive abilities

**Figure 2:**
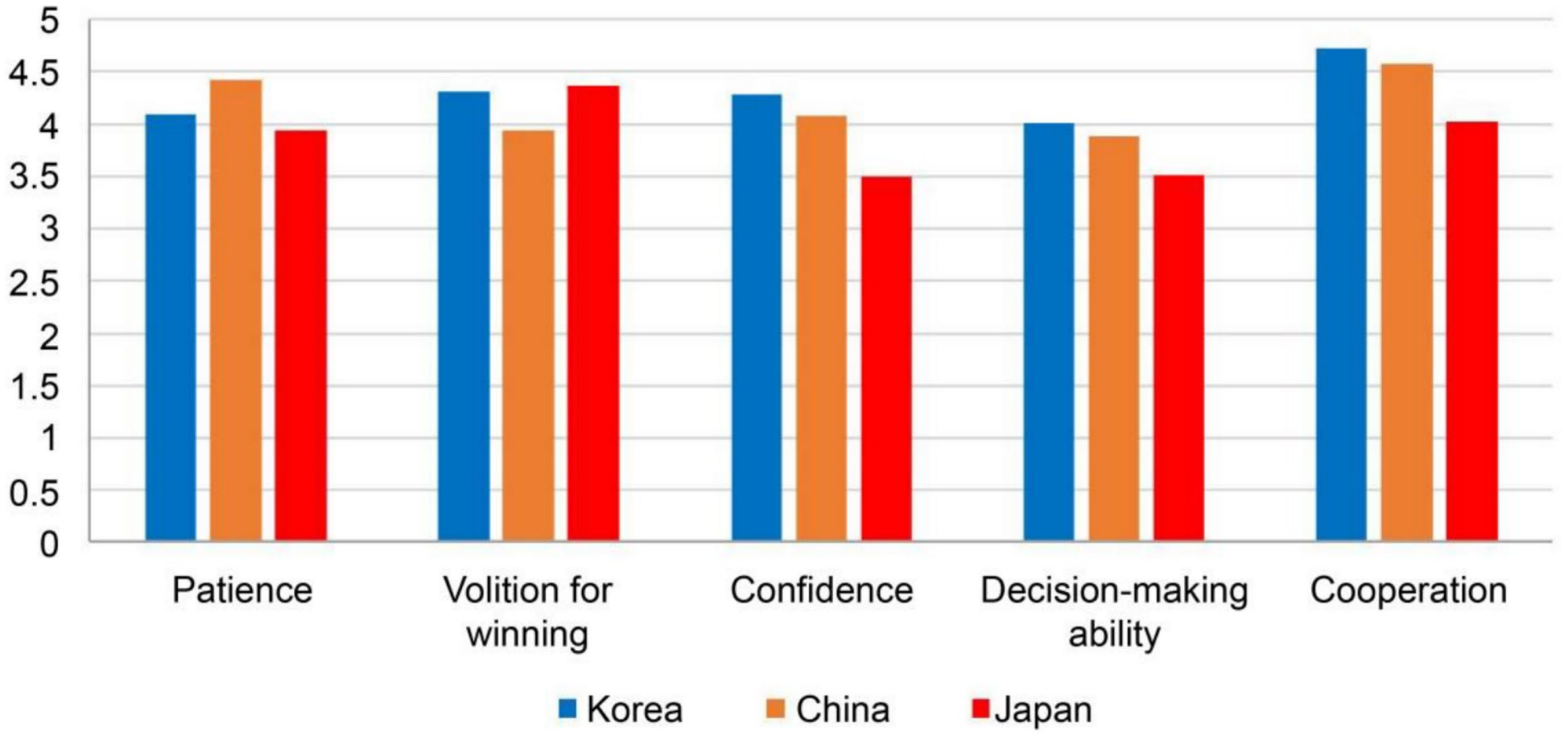
Comparison of five psychological competitive abilities among South Korean, Chinese, and Japanese youth soccer players.

Psychological competitive abilities were analyzed across the three countries using ANCOVA, controlling for age. Age did not significantly influence patience (*F*(1,462) = 0.095, *p* = 0.758), volition for winning (F(1,462) = 0.257, *p* = 0.612), confidence (F(1,462) = 0.083, *p* = 0.773), decision-making ability (F(1,462) = 2.205, *p* = 0.138), or cooperation (F(1,462) = 0.010, *p* = 0.920). Significant main effects of country were observed for all five abilities, namely patience (*F*(2,462) = 21.302, *p* < 0.001, η^2^ = 0.084), volition for winning (F(2,462) = 20.837, *p* < 0.001, η^2^ = 0.083), confidence (F(2,462) = 59.913, *p* < 0.001, η^2^ = 0.206), decision-making ability (F(2,462) = 24.108, *p* < 0.001, η^2^ = 0.094), and cooperation (F(2,462) = 69.932, *p* < 0.001, η^2^ = 0.232). Post-hoc comparisons revealed that Chinese players scored highest on patience and decision-making ability, while South Korean and Japanese players scored higher on volition for winning than Chinese players. South Korean players showed the highest levels of confidence and cooperation. These results indicate that the observed cross-national differences were robust and independent of age (Table 3; Figure 2).

**Table 3:** ANCOVA results for key psychological competitive abilities, controlling for age.

Dependent variable (DV)	Covariate age F (p)	Country main effect F (p)	Partial η^2^	Adjusted means (EMM) South Korea / China / Japan	*Post-hoc* Comparison
Patience	0.095 (0.758)	21.302 (<0.001)	0.084	4.09/4.42/3.95	China > South Korea, Japan
Volition for Winning	0.257 (0.612)	20.837 (<0.001)	0.083	4.32/3.94/4.37	South Korea, Japan > China
Confidence	0.083(0.773)	59.913 (<0.001)	0.206	4.30/4.08/3.50	South Korea > China > Japan
Decision-making Ability	2.205(0.138)	24.108(<0.001)	0.0.94	3.88/4.02/3.51	China > South Korea, Japan
Cooperation	0.010 (0.920)	69.932(<0.001)	0.232	4.73/4.73/4.02	South Korea, China > Japan

EMM, Estimated Marginal Means, adjusted for age; n.s., not significant. *Post-hoc* comparisons were conducted using Bonferroni correction. ***p < 0.001.

## 4 Discussion

All comparisons were conducted using ANCOVA with age as a covariate, ensuring that the observed cross-national differences were independent of age. This study aimed to provide foundational data for developing training and game strategies for coaches by analyzing and comparing the psychological competitive abilities of South Korean, Chinese, and Japanese youth soccer players.

Understanding athletes’ psychological capabilities is crucial for predicting future performance (Feichtinger and Höner, 2014; Höner and Feichtinger, 2016; Musculus and Lobinger, 2018; Vaeyens et al., 2008), and the importance of psychological competitive abilities has been increasingly emphasized in modern soccer training (Musculus and Lobinger, 2018; Raglin, 2001; Vestberg et al., 2012). Youth athletes’ psychological traits are particularly critical for their successful transition to adult-level performance; therefore, assessing these abilities is important for effective coaching and designing game strategies (Abdullah et al., 2016; Morris, 2000).

In this study, we compared the psychological competitive abilities of youth soccer players from three Northeast Asian countries that are currently investing significant resources into youth soccer development. Understanding these abilities can help build a strong foundation for athlete growth, and cross-national comparisons provide valuable data, as discussed in the following sections. The hypotheses of this study are partially supported.

### 4.1 Differences in patience

Chinese athletes demonstrated significantly higher levels of patience than their South Korean and Japanese counterparts. This finding aligns with the cultural emphasis on persistence and endurance often observed in Chinese sports training programs, as supported by the national model of centralized sports development (Kim and Chung, 2007). Coaches working with Chinese athletes can leverage this trait by incorporating more long-term, endurance-based drills that emphasize consistent performance under pressure. For example, training sessions that simulate extended, high-stress competitive environments could help enhance athletes’ mental resilience, enabling them to remain focused during protracted matches.

In contrast, South Korean and Japanese athletes exhibited comparatively lower levels of patience. Coaches in these countries might consider introducing activities that encourage sustained attention and mental endurance. Practices could include high-intensity drills that span longer periods, challenging athletes to maintain focus, especially under fatigue. According to Beilock and Gray (2007), fostering patience in athletes through targeted interventions can positively impact their overall psychological readiness in high-stakes situations, such as penalty shootouts or the final stages of a match.

Importantly, our results both resonate with and diverge from prior research. In line with Abdollahi Dehkordi and Taheri (2025), who emphasized that patience reduces impulsivity and enhances emotional regulation in athletes, our findings underscore mental stability as a crucial factor in sports performance. Conversely, Vaughan et al. (2021) found that athlete expertise—not cultural background—was the primary predictor of improved reflection-impulsivity (a concept closely related to patience), suggesting that certain cognitive traits may be more universal than culturally specific. This contrast highlights the need for culture-sensitive training approaches while also recognizing the role of general expertise development.

### 4.2 Differences in volition for winning

South Korean and Japanese youth soccer players demonstrated higher volition for winning than their Chinese counterparts. This motivation is often seen as a key psychological trait that drives athletes to continuously push their limits in competition (Lal, 2006). In the context of South Korean athletes, this heightened drive for victory may be linked to the competitive pressure imposed by societal norms and the strong emphasis on winning (Lee and Kim, 2005). Coaches working with South Korean and Japanese players should capitalize on this strong desire to win by setting high, yet attainable, performance goals. Training that encourages a growth mindset—where effort and persistence are rewarded—may enhance this intrinsic motivation, aligning with recent findings by Hollenbeck and Hall (2004), which suggest that motivation is crucial for performance enhancement.

However, this desire to win can also have negative effects. Excessive volition for winning can lead to higher competitive anxiety, as seen in athletes who may feel pressured to constantly achieve success (Jamshidi et al., 2011). Coaches should be mindful of this potential downside and introduce strategies to moderate this trait, such as relaxation techniques or cognitive–behavioral training aimed at managing pressure (Swann et al., 2017). In addition, athletes should be taught how to manage setbacks, ensuring that their desire to win does not negatively impact their mental health or decision-making on the field.

Importantly, our results align with the findings of Lee et al. (2021), who found that while South Korean adolescent athletes displayed high achievement goal orientation, their confidence was closely tied to their perception of error feedback from coaches—highlighting a blend of intrinsic drive supported by extrinsic evaluation. This underscores the importance of balancing goal orientation with adaptive feedback systems, ensuring that athletes’ volition for winning remains motivating without increasing anxiety.

### 4.3 Differences in confidence

Confidence is a key factor in athletes’ performance, directly affecting their competitive behavior and psychological state. South Korean athletes exhibited the highest level of confidence, followed by Chinese athletes, with Japanese athletes showing the lowest levels. In China, the cultural context shaped by Confucian social values fosters a deep sense of trust and deference toward authority figures, including coaches (Tang et al., 2023; Zhu J. et al., 2024; Zhu R. et al., 2024). As a result, Chinese athletes often perceive even strict coaching as benevolent guidance, maintaining stable and trusting relationships with their coaches—an effect reinforced by the Confucian concept of Guanxi, which emphasizes loyalty and reciprocity (Tang et al., 2023). In South Korea, family support notably enhances athlete confidence through parental encouragement and social validation (Choi, 2016; Gao et al., 2024).

Moreover, Coussens et al. (2025) highlighted that perceived coach support significantly predicts self-confidence, emphasizing that the quality of coach–athlete relationship is critical for psychological development. Chinese athletes’ higher baseline confidence can therefore be attributed to both familial influences and culturally embedded, trust-based coach–athlete relationships. Japanese athletes with lower confidence levels may benefit from personalized intervention strategies such as goal setting, self-talk, and imagery (Hollenbeck and Hall, 2004; Vealey, 1986).

In summary, familial, cultural, and coach–athlete relationship dynamics jointly influence athlete confidence, suggesting that culturally informed coach support can consolidate and boost confidence effectively.

### 4.4 Differences in decision-making ability

In terms of decision-making ability, Chinese and South Korean athletes demonstrated higher performance than Japanese athletes. This difference may reflect variations in training philosophies: South Korean football emphasizes quick-reaction drills (Otake et al., 2006), China uses stress-simulation training within its centralized system (Kim and Chung, 2007), and Japan focuses more on technical precision (Vestberg et al., 2012). Coaches should therefore design tailored programs that integrate cognitive–behavioral and situational training to enhance both decision-making speed and accuracy (Landers, 1980; Zhu J. et al., 2024; Zhu R. et al., 2024).

Consistent with our findings, Mann et al. (2007) conducted a meta-analysis of perceptual–cognitive expertise in sports, showing that expert athletes consistently outperform novices in decision accuracy and response time due to more efficient visual search strategies and information-processing patterns.

Furthermore, Zhu J. et al. (2024), in their systematic review, confirmed that perceptual–cognitive training positively enhances both anticipation and decision-making skills in team sports, although transfer to actual game performance remains moderate.

These findings suggest that expertise development—achieved through perceptual–cognitive drills—is central to enhancing decision-making across cultures. Training should therefore emphasize both high-level skill development and cultural suitability to foster consistent athlete performance.

### 4.5 Differences in cooperation

Cooperation is a crucial psychological trait in team sports. South Korean athletes showed the highest level of cooperation, followed by Chinese athletes, with Japanese athletes performing relatively worse.

This difference aligns with South Korea’s strong collectivist cultural orientation, which emphasizes teamwork and shared goals. Indeed, organizational research shows that in collectivist societies such as Korea, clan-like or collaborative cultural practices significantly boost group cohesion and team performance (Park and Fritz, 2021).

In athletic settings, Song and Xue (2022) demonstrated that, in Chinese team sports, psychological collectivism mediates the relationship between cohesion and athlete engagement. This suggests that cultural values shape cooperative behavior through internalized group loyalty.

Therefore, South Korean coaches should emphasize team-building exercises, incorporating small-sided, cooperative drills to enhance trust, coordination, and collective efficacy among players.

Chinese coaches, while also operating within a collectivist framework, may benefit from more structured cooperation training. Implementing paired or small-group tactical drills can foster interdependence, cultivating cooperation and team spirit in line with national sports goals (Tian et al., 2015).

For Japanese athletes, whose cooperation was relatively weaker, coaches can strengthen teamwork by focusing on collective goal-setting and clearly defining team tasks, helping athletes recognize the importance of collaboration for competitive success (Daniels, 2007).

Importantly, cohesion is generally linked to better team performance across cultures (Beal et al., 2003; Carron et al., 2002). Including culturally informed cohesion-building strategies—from socio-emotional bonding activities to task-oriented drills—can maximize cooperative potential and enhance competitive outcomes across these East Asian contexts.

## 5 Concentration and limitations

This study aimed to provide foundational data for developing effective training and match strategies by comparing the psychological competitive abilities of youth soccer players from South Korea, China, and Japan. Among the five key psychological constructs examined—patience, volition for winning, confidence, decision-making ability, and cooperation—statistically significant differences were observed. These traits are widely recognized as critical for athletic performance under pressure, influencing motivation, focus, and interpersonal functioning during competition (Vealey, 1986; Hollenbeck and Hall, 2004; Swann et al., 2017). By identifying cultural and developmental patterns in these traits, our findings offer meaningful guidance for coaches to create training environments based on athletes’ psychological profiles.

However, several limitations must be acknowledged when interpreting the results.

First, although this study highlights cross-cultural differences in psychological competencies, these differences do not directly translate into competitive performance. Athletic success is determined by a complex interplay of physical ability, technical skill, and mental preparedness (Zhu J. et al., 2024; Zhu R. et al., 2024). Consequently, psychological traits, although influential, should be interpreted as part of a broader performance ecosystem.

Second, this study did not incorporate physiological or anthropometric variables such as BMI, biological maturity, or playing position due to methodological constraints. These factors can significantly influence the development and expression of psychological skills. Future studies should adopt a mixed methods approach, combining psychometric tools, such as the DIPCA.3, with physical performance data to provide a more comprehensive understanding of youth athletes’ profiles.

Third, although statistical differences were identified, the underlying mechanisms driving cross-cultural variations remain unclear. Variables such as coaching philosophy, athlete socialization, and training context may contribute to these outcomes. Therefore, future research should incorporate qualitative methods, such as in-depth interviews with athletes, coaches, and sports psychologists, to investigate the contextual and cultural foundations of these psychological traits. In addition, using structural equation modeling (SEM) could be valuable for testing theoretical models and causal pathways.

Furthermore, several DIPCA.3 sub-factors—including aggressiveness, self-control, concentration, ability to relax, and predictive judgment—did not differ significantly among the countries. This may reflect a common baseline of psychological development in structured youth football programs throughout East Asia or it may indicate limitations in the instrument’s sensitivity to subtle cultural nuances. Given the scarcity of comparative research on these dimensions, further targeted studies are needed to determine whether such similarities are meaningful or artifacts of methodological design.

Finally, the generalizability of these findings is limited by the lack of detailed sample descriptors. Important variables, such as athletes’ years of playing experience, regional training environments, and level of competition, were not fully reported. In cross-national research, transparent and comprehensive sample characterization is essential to ensure that the findings are both reliable and applicable to broader populations. Future studies should prioritize this aspect to strengthen the validity of international comparisons.

## Data Availability

The raw data supporting the conclusions of this article will be made available by the authors, without undue reservation.
